# Supplementary material for: Horsenettle (Solanum carolinense) fruit bacterial communities are not variable across fine spatial scales
Source: PeerJ. 2021 Nov 8;9:e12359. doi: 10.7717/peerj.12359 (PMC8582302; doi:10.7717/peerj.12359)
Supplement: Supplemental Information 1 — Information on sample name, latitude, longitude, and number of samples collected at a site. [file peerj-09-12359-s001.docx]

| Site info | latitude | longitude | # samples |
| --- | --- | --- | --- |
| Site 1 | 37.237595 | -80.45429 | 2 |
| Site 2 | 37.23814 | -80.45244 | 3 |
| Site 3 | 37.23958 | -80.454895 | 2 |
| Site 4 | 37.239548 | -80.45639 | 4 |
| Site 5 | 37.240814 | -80.45765 | 2 |
| Site 6 | 37.239967 | -80.45996 | 3 |
| Site 7 | 37.24023 | -80.46111 | 5 |
| Site 8 | 37.240257 | -80.461105 | 2 |

**Supplemental Table 1: Sample information**. Information on sample name, latitude, longitude, and number of samples collected at a site.
