# Supplementary material for: Horsenettle (Solanum carolinense) fruit bacterial communities are not variable across fine spatial scales
Source: PeerJ. 2021 Nov 8;9:e12359. doi: 10.7717/peerj.12359 (PMC8582302; doi:10.7717/peerj.12359)
Supplement: Supplemental Information 2 — Primer sequences used for locked nucleic acid PCR (LNA) and 16s rRNA PCR. [file peerj-09-12359-s002.docx]

| **LNA oligonucleotide Primer Sequence** | |
| --- | --- |
| 63F-mod | 5'-YRKGCYT WAYACATGCAAGTC-3' |
| 1492r | 5’-GGYTACCTTGTTACGACTT-3’ |
| LNA-Mit63 | 5'-GTCGAACGTTGTTTTCGGp-3' |
| LNA-Mit1492 | 5'-CTTCACCCCAGTCGAAGAp-3' |
| LNA-Pla63c | 5'-TCGGACGGGAAAACACG-3' |
| LNA-Pla1492b | 5'-CTTCACTCCAGTCACTAGC-3' |
|  |  |
|  |  |
| **16s rRNA V4 Primer Sequence** | |
| 515F | 5'-GTGCCAGCMGCCGCGGTAA-3' |
| 806R | 5'-GGACTACHVHHHTWTCTAAT-3' |

**Supplemental Table 2. LNA and 16s rRNA V4 region primer sequences**. Primer sequences used for locked nucleic acid PCR (LNA) and 16s rRNA PCR.
