# Supplementary material for: Horsenettle (Solanum carolinense) fruit bacterial communities are not variable across fine spatial scales
Source: PeerJ. 2021 Nov 8;9:e12359. doi: 10.7717/peerj.12359 (PMC8582302; doi:10.7717/peerj.12359)
Supplement: Supplemental Information 3 [file peerj-09-12359-s003.docx]

| Sample | input | filtered | denoised | tabled | nonchim |
| --- | --- | --- | --- | --- | --- |
| 21 | 44604 | ﻿38896 | 38278 | 38278 | ﻿37429 |
| 27 | 29330 | 25527 | ﻿25337 | 25337 | 25111 |
| 30 | 40207 | 35025 | 34809 | ﻿34809 | ﻿ 34514 |
| 33 | 32220 | ﻿28655 | ﻿27635 | 27635 | 27244 |
| 34 | 37766 | 33098 | 32467 | 32467 | 31673 |
| 35 | 17109 | ﻿15411 | 14990 | 14990 | 14754 |
| 36 | 20804 | 17819 | 17690 | 17690 | ﻿17375 |
| 37 | 23878 | 20967 | ﻿20703 | 20703 | 20478 |
| 38 | 20183 | ﻿ 17869 | 17443 | 17443 | ﻿ 17146 |
| 39 | 17163 | ﻿15334 | ﻿15066 | 15066 | ﻿14940 |
| 42 | 31325 | 27993 | ﻿27671 | 27671 | 26648 |
| 43 | 30950 | ﻿27621 | 27354 | 27354 | 26808 |
| 45 | 37298 | ﻿33459 | 32913 | 32913 | 31517 |
| 50 | 33690 | ﻿ 30379 | 29787 | 29787 | 28868 |
| 53 | 48585 | 42698 | ﻿42156 | 42156 | 40739 |
| 61 | 49643 | 47031 | 46809 | 46809 | ﻿43551 |
| 62 | 76647 | 71024 | ﻿ 70254 | 70254 | 68397 |
| 64 | 73234 | ﻿ 69179 | 68622 | 68622 | ﻿ 53473 |
| 65 | 115900 | 107512 | 106423 | 106423 | ﻿99872 |
| 66 | 82480 | ﻿76745 | ﻿76094 | 76094 | 74254 |
| 67 | 77960 | ﻿73128 | ﻿72226 | 72226 | ﻿67331 |
| 68 | 81348 | ﻿76603 | 75965 | 75965 | ﻿67165 |
| 69 | 83648 | ﻿77923 | 77264 | 77264 | 76301 |

**Supplemental Table 3. Sample information and reads**. Sample information and reads input, filtered, denoised, tabled, and nonchim.
