# Supplementary figures and images for: Horsenettle (Solanum carolinense) fruit bacterial communities are not variable across fine spatial scales
Source: PeerJ. 2021 Nov 8;9:e12359. doi: 10.7717/peerj.12359 (PMC8582302; doi:10.7717/peerj.12359)

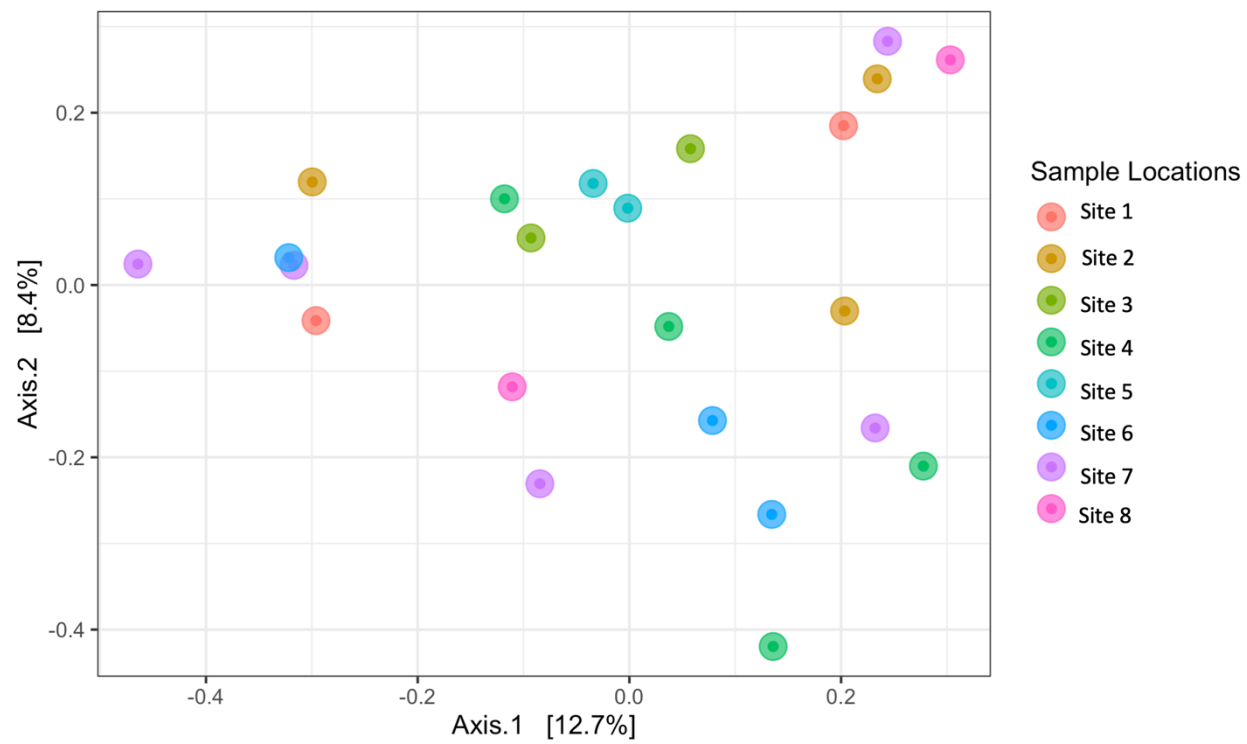

Supplement: Supplemental Information 4 — PCoA plot was used to determine differences between microbial communities associated with S. carolinense fruit and sample locations (PERMANOVA, p = 0.688, R2 = 0.30896). The axes indicate the percentage of variation in the data with axis 1 (the first component) representing 12.7% of the variation and axis 2 (the second component) representing 8% of the variation. [file peerj-09-12359-s004.pdf]
