# Supplementary material for: Horsenettle (Solanum carolinense) fruit bacterial communities are not variable across fine spatial scales
Source: PeerJ. 2021 Nov 8;9:e12359. doi: 10.7717/peerj.12359 (PMC8582302; doi:10.7717/peerj.12359)
Supplement: Supplemental Information 5 — Statistical outputs for (A) observed richness, Faith’s phylogenetic diversity, (B) Jaccard index, (C) Bray–Curtis, and (D) Mantel. [file peerj-09-12359-s005.pdf]

a.

```
        Kruskal-Wallis rank sum test

data:  alpha_tests$Observed and alpha_tests$pop
Kruskal-Wallis chi-squared = 9.5226, df = 7, p-value = 0.2173


        Kruskal-Wallis rank sum test

data:  alpha_tests$PD and alpha_tests$pop
Kruskal-Wallis chi-squared = 10.012, df = 7, p-value = 0.1879
```

b.

```
Call:
adonis(formula = Jaccard_site ~ pop_ID, data = sampledf_site)

Permutation: free
Number of permutations: 999

Terms added sequentially (first to last)

          Df SumsOfSqs MeanSqs F.Model      R2 Pr(>F)
pop_ID     7    2.7043 0.38633 0.95808 0.30896  0.688
Residuals 15    6.0485 0.40323         0.69104
Total     22    8.7528                 1.00000
```

c.

```
Call:
adonis(formula = bray_site ~ pop_ID, data = sampledf_site)

Permutation: free
Number of permutations: 999

Terms added sequentially (first to last)

          Df SumsOfSqs MeanSqs F.Model     R2 Pr(>F)
pop_ID     7    2.2042 0.31489 0.93286 0.3033  0.711
Residuals 15    5.0633 0.33755         0.6967
Total     22    7.2675                 1.0000
```

d.

```
Mantel statistic based on Pearson's product-moment correlation

Call:
mantel(xdis = dist.matrix.scaro, ydis = bray_site, method = "pearson",      permutations = 999, strata = NULL,
 na.rm = TRUE, parallel = getOption("mc cores"))

Mantel statistic r: 0.03343
      Significance: 0.302

Upper quantiles of permutations (null model):
   90%    95%  97.5%    99%
0.0907 0.1186 0.1428 0.1781
Permutation: free
Number of permutations: 999
```
